# Short-term health-related quality of life and symptom control with docetaxel, cisplatin, 5-fluorouracil and cisplatin (TPF), 5-fluorouracil (PF) for induction in unresectable locoregionally advanced head and neck cancer patients (EORTC 24971/TAX 323)

**DOI:** 10.1038/sj.bjc.6605860

**Published:** 2010-09-14

**Authors:** C M L van Herpen, M E Mauer, R Mesia, M Degardin, S Jelic, C Coens, J Betka, J Bernier, E Remenar, J S Stewart, J H Preiss, D van den Weyngaert, A Bottomley, J B Vermorken

**Affiliations:** 1 Department of Medical Oncology, Radboud University Nijmegen Medical Centre, 452, PO Box 9101, 6500 HB Nijmegen, The Netherlands; 2 European Organisation for Research and Treatment of Cancer, EORTC Headquarters, Brussels, Belgium; 3 Institut Catala d'Oncologia, Barcelona, Spain; 4 Centre Oscar Lambret, Lille, France; 5 The Institute of Oncology and Radiology, Belgrade, Serbia; 6 University Hospital Motol, Prague, Czech Republic; 7 Clinique de Genolier, Genolier, Switzerland; 8 National Institute of Oncology, Budapest, Hungary; 9 Charing Cross Hospital, London, United Kingdom; 10 Caritasklinik St Theresia, Saarbrucken, Germany; 11 Ziekenhuis Netwerk Antwerpen Middelheim, Antwerp, Belgium; 12 Universitair Ziekenhuis Antwerpen, Edegem, Belgium

**Keywords:** HRQOL, symptoms, head and neck cancer

## Abstract

**Background:**

The EORTC 24971/TAX 323, a phase III study of 358 patients with unresectable locoregionally advanced squamous cell carcinoma of the head and neck, showed an improved progression-free and overall survival (OS) with less toxicity when docetaxel (T) was added to cisplatin and 5-fluorouracil (PF) for induction and given before radiotherapy (RT). The impact of the addition of docetaxel on patients’ health-related quality of life (HRQOL) and symptoms was investigated.

**Methods:**

HRQOL was assessed at baseline, at end of cycle 2, and 4, 6, and 9 months after completion of RT using the European Organisation for Research and Treatment of Cancer (EORTC) Quality of Life Questionnaire C30 (QLQ-C30) and the EORTC QLQ Head and Neck Cancer-Specific Module (EORTC QLQ-H&N35). The primary HRQOL scale was global HRQOL per protocol.

**Results:**

Compliance to HRQOL assessments was 97% at baseline, but dropped to 54% by 6 months. Data were analysed up to 6 months. There was a trend towards improved global HRQOL during the treatment period. At 6 months after the end of RT, global HRQOL was higher in the TPF arm than in the PF arm, but the low compliance does not allow to draw definitive conclusions. Swallowing and coughing problems decreased more in the TPF arm than in the PF arm at the end of cycle 2, but to a limited extent.

**Conclusion:**

Induction chemotherapy with TPF before RT not only improves survival and reduces toxicity compared with PF but also seems to improve global HRQOL in a more sustainable manner.

Head and neck cancer mostly influences health-related quality of life (HRQOL) in a negative manner and can induce symptoms, which may interfere with daily life. Both disease and treatment can affect important functions such as eating, swallowing, and speaking, as well as the physical appearance (Michiwaki *et al*, 1992; Pauloski *et al*, 1994; Argiris *et al*, 2008). Acute side-effects related to treatment of head and neck cancer may include pain, dermatitis, mucositis, dysphagia, and anorexia (Dropkin, 1998; Pauloski *et al*, 1998; Chaplin and Morton, 1999; Rosenthal *et al*, 2006). Some late complications, such as xerostomia, may persist for a long time and may even be permanent, having an adverse effect on patient HRQOL and delaying or preventing resumption of normal activities (Nguyen *et al*, 2005; Bentzen and Trotti, 2007). In addition, a local or locoregional recurrence occurring after an intensive primary treatment has great impact on HRQOL.

We recently reported clinical results from our phase III study, EORTC 24971/TAX 323 (Vermorken *et al*, 2007). In the treatment of unresectable locoregionally advanced squamous cell carcinoma of the head and neck (SCCHN) of the oral cavity, oropharynx, hypopharynx, or larynx, the combination of docetaxel, cisplatin, and 5-fluorouracil (TPF) proved to be significantly more effective than the standard Wayne-State University cisplatin and infusional 5-fluororuacil (PF) regimen when given as induction chemotherapy (CT) before radiotherapy (RT) (Vermorken *et al*, 2007). A total of 358 patients underwent randomisation, with 177 assigned to the TPF group and 181 to the PF group. At a median follow-up of 32.5 months, the median progression-free survival was 11.0 months in the TPF group and 8.2 months in the PF group (hazard ratio for disease progression or death in the TPF group, 0.72; *P*=0.007). Treatment with TPF resulted in a reduction in the risk of death of 27% (*P*=0.02), with a median overall survival (OS) of 18.8 months, as compared with 14.5 months in the PF group. There were more grade 3 or 4 events of leucopenia and neutropenia in the TPF group and more grade 3 or 4 events of thrombocytopenia, nausea, vomiting, stomatitis, and hearing loss in the PF group. In this paper, we report the analysis of HRQOL and symptoms.

## Methods

### Study design and treatment

This international multi-centred European Organisation for Research and Treatment of Cancer (EORTC) study randomly assigned patients to either the control arm with cisplatin (100 mg m^−2^) administered as a 1-h IV infusion on day 1 followed by the continuous infusion of 5-FU (1000 mg m^−2^ per day) from day 1 to day 5, or the experimental arm with docetaxel (75 mg m^−2^) administered as a 1-h IV infusion on day 1 followed by cisplatin (75 mg m^−2^) given over 1 h by IV infusion on day 1 and then starting the continuous IV infusion of 5-FU (750 mg m^−2^ per day) from day 1 to day 5. Treatment was administered every 3 weeks (defined as one cycle) for up to four cycles, unless progressive disease, unacceptable toxicity, or patient refusal occurred, whatever came first. Thereafter, patients had to receive RT, which was delivered during a 7-week period with the use of either conventional fractionation (total dose, 66–70 Gy) or accelerated or hyperfractionated regimens (total maximum dose of 70 Gy for the accelerated regimen and 74 Gy for the hyperfractionated regimen). The majority of patients received conventional RT. All patients were assessed by a head and neck surgeon before start of CT and after RT. If a neck dissection was advised, it was performed 3 months after the completion of RT.

The primary end point of the study was progression-free survival; secondary end points included response rate before and after RT, duration of response, time to treatment failure, OS and HRQOL. Full details of the clinical results were reported in Vermorken *et al* (2007). The trial, approved by the EORTC protocol review committee and the ethics committee of each participating centre, was conducted in accordance with the Helsinki Declaration. All patients provided written informed consent before randomisation. Randomisation was done centrally at the EORTC headquarters, Belgium, using a minimisation technique. Randomisation was balanced according to the primary tumour site (oral cavity, oropharynx, hypopharynx, or larynx) and the centre.

### Procedures for QOL data collection

The EORTC QOL Questionnaire C30 (EORTC QLQ-C30, version 3) was selected as it is a robust validated tool and the one that is most frequently used in randomised clinical trials (Aaronson *et al*, 1993; Garratt *et al*, 2002). The EORTC QLQ-C30 measure comprises five functioning scales: physical, role, emotional, cognitive, and social; three symptom scales: fatigue, nausea/vomiting, and pain; six single item scales: dyspnoea, sleep disturbance, appetite loss, constipation, diarrhoea, and financial impact; and the Global HRQOL scale. The items on the measures were scaled and scored using recommended EORTC procedures, with a higher score representing a higher level of functioning or higher level of symptoms (Fayers *et al*, 1999).

In addition, given that the problems experienced by patients with head and neck cancer may not be fully addressed by the EORTC QLQ-C30, the EORTC Head and Neck module, the EORTC QLQ-H&N35, with 35 items specifically developed for head and neck patients in cancer clinical trials, was included (Bjordal *et al*, 2000). This measure has a structure of seven symptom scales (pain, swallowing, senses, speech, social eating, social contact, and sexuality), six symptom items (problems with teeth, problems with opening mouth, dry mouth, sticky saliva, coughing, and feeling ill), and five additional items related to the use of painkillers, nutritional supplements and feeding tube, and changes in body weight.

Furthermore, the clinician-assessed Performance Status Scale for Head and Neck Cancer Patients (PSS-HN) tool, containing three questions on eating in public, understandability of speech, and normalcy of diet, was used, as this can provide unique information, independent of HRQOL measures (List *et al*, 1996). A validated visual analogue scale *ad hoc* pain thermometer was also employed.

As per protocol, the HRQOL questionnaires had to be completed before knowledge of treatment allocation by the patient (up to 2 weeks before randomisation), at cycle 2 just before the next cycle (at the time of tumour assessment), at the end of CT before starting RT (at the time of tumour assessment), and then, 6 and 9 months after completion of RT. Patients were asked to complete the questionnaires regardless of stable or progressive disease or relapse. Guidelines for administering questionnaires were provided, ensuring standardisation of HRQOL data by all personnel (Young *et al*, 2002). The two EORTC measures were translated and culturally validated before use in this study, in accordance with standard EORTC practices.

### Statistical analysis

HRQOL was a secondary study end point, whereas the sample size calculation was based on assessment of the primary end point (PFS). To reduce multiple testing, five primary domains were preselected for this trial: global HRQOL from the EORTC QLQ-C30, and pain, swallowing, speech, and coughing from the EORTC QLQ-H&N35 module. On the basis of results of a phase II study showing that patients treated with TPF had a rapid and substantial tumour shrinkage, which may result in an improvement of the local symptoms commonly reported with locally advanced tumours (Schrijvers *et al*, 2004), it was anticipated that the experimental arm (TPF) would be superior to the control arm (PF) in lowering symptom levels. Owing to the expected higher toxicity of the experimental arm, no significant difference in the global score during treatment was anticipated. The remaining HRQOL and symptom variables were then examined on an exploratory basis. The results of this study are presented in accordance with recent criteria for reporting HRQOL (Efficace *et al*, 2003).

All analyses were performed using SAS version 9.1.3 (Licence through EORTC), according to the intent-to-treat principle. All patients were analysed in their assigned treatment arm.

For the purpose of the analysis, time windows for accepting HRQOL forms were defined as follows: baseline HRQOL assessments had to be obtained no more than 14 days from randomisation and before the start of CT; HRQOL assessments at the end of cycles 2 and 4 had to be obtained no more than 3 weeks from the end of cycle 2 or 4 and before the start of RT; HRQOL assessments at 6 months after RT had to be obtained no sooner than 3 months after the end of RT and no more than 7.5 months after the end of RT.

A mixed model with an undefined covariance structure was fitted to the longitudinal HRQOL data (for each selected score) to test for differences between the two treatment arms. All patients with at least one valid HRQOL form were included in the analysis (*n*=353).

Using a standard established method of interpretation for the HRQOL scores for the EORTC tool, the minimal important difference was calculated (Osoba *et al*, 1998). Differences of at least 10 points (on a 0–100 scale) were classified as the minimum clinically meaningful change in a HRQOL parameter. For claims of potential improvement in the five selected scales, the level of statistical significance was fixed at 0.01 to reduce the risk of false-positive findings. However, the adjustment of the *P*-value was only partial, as it did not take into account multiple testing due to multiple time points.

Given that missing data is a common problem in HRQOL studies and could bias the results, compliance rates with per-protocol HRQOL assessments were compared between treatment arms, and the missingness mechanism was graphically investigated. Compliance was computed as the number of received QoL forms divided by the number of expected forms at each time point. Expected forms were for patients still on treatment at the given time point (i.e., for patients who started cycle 2 at the time point ‘End of cycle 2’, cycle 4 at the time point ‘End of cycle 4’, and RT at the time point ‘At 6 months after RT’). Complementary analyses of the proportion of patients experiencing a worsening or an improvement of more than 10 and 20 points (respectively classified as moderate and large effects), were performed as sensitivity analyses (Osoba *et al*, 1998). As QoL scales only take a finite number of equidistant values from 0 to 100, that is, for the Global HRQOL scale, 0, 8.3, 16.6, 25, and so on, a worsening or an improvement of 10 points and 20 points will correspond to a 16.6 and 25 points difference, respectively.

## Results

Between April 1999 and March 2002, 358 patients from 37 institutions in 15 European countries were randomised between the TPF arm (177) and the PF arm (181). The two treatment arms were balanced according to baseline demographic and clinical characteristics (Vermorken *et al*, 2007).

### HRQOL: compliance and baseline scores

QoL data from 353 patients among a total of 358 patients (99%) were included in the analysis. Overall compliance to the QLQ-C30 questionnaire was 97% at baseline, 86% at the end of cycle 2, and 76% at the end of cycle 4 (Table 1). Compliance was above 50% at 6 months after RT (108 patients). Because the compliance dropped below 50% at 9 months after RT, data were analysed up to 6 months after RT. Fisher exact tests for compliance difference between the two treatment arms revealed no significant difference at baseline, nor at any follow-up time points (Table 1).

Although there were very few missing scores for the EORTC QLQ-C30 questionnaire, the number of missing items for the head and neck module was higher. Specifically, 35% of the planned HRQOL assessments did not include the head and neck module because at the time of the trial, validated and translated questionnaires were not available in countries such as the Czech Republic, Hungary, Serbia, and Slovak Republic. For the head and neck module, no interpretation of the results was made at 6 months after RT because of the very low amount of data collected.

For each of the preselected HRQOL scales, global HRQOL, pain, swallowing, speech, and coughing, the evolution of the mean scores just before missingness was graphically investigated to check the validity of the mixed model. A sharp increase or decrease in scores just before missingness is usually a good indicator of non-ignorable missing data. None of our analyses indicated a possible non-ignorable missingness process.

Baseline HRQOL scores were quite similar in both treatment arms and comparable with the reference values in head and neck cancer patients with stage III–IV disease (Table 2) (Scott *et al*, 2008).

### Primary HRQOL scales: between arm differences and changes over time

**Figure 1:**
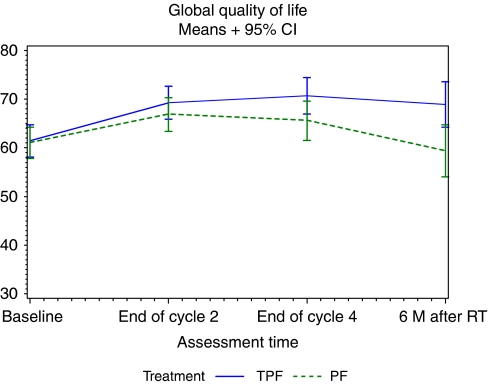
Evolution of mean scores in global QoL over time.

Global HRQOL increased during CT in both treatment arms and was maintained at 6 months after the end of RT in the TPF arm while it decreased in the PF arm (Table 3, Figure 1). The mean difference between the treatment arms at 6 months after the end of RT was 9.5 points, very close to the clinically meaningful change of 10 points and statistically significant (*P*=0.0092). However, when analysing the change from baseline, the *P*-value was reduced to 0.0211, not below the statistical level of significance of 0.01.

**Figure 2:**
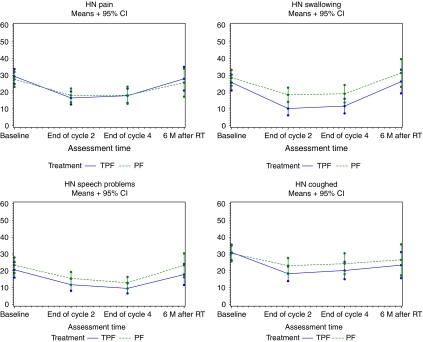
Evolution of mean scores in other selected scales over time.

Pain, swallowing problems, speech problems, and coughing decreased during CT in both treatment arms (Figure 2). There were no treatment-related differences in pain and speech problems. Swallowing and coughing problems decreased more in the TPF arm compared with the PF arm. The differences between the two treatment arms did not reach the 10-point difference at any time point. When analysing changes from baseline, no difference was statistically significant.

### Sensitivity analyses

The percentage of patients who experienced either a moderate (>10 points) or important (>20 points) worsening/improvement in each selected scale at any of the three time points (end of cycle 2, end of cycle 4 and 6 months after the end of RT), compared with baseline, were compared between treatment arms. These additional analyses confirmed the overall trend towards an advantage of the TPF arm compared with the PF arm with a higher percentage of patients experiencing an improvement and a lower percentage of patients experiencing a worsening in global HRQOL, coughing, and swallowing. For pain and speech problems, the percentage of patients worsening in the TPF arm was higher, but the percentage showing an improvement was also higher. The differences in proportions did not exceed 10% and were not statistically significant (Table 4).

### Other HRQOL scales

**Figure 3:**
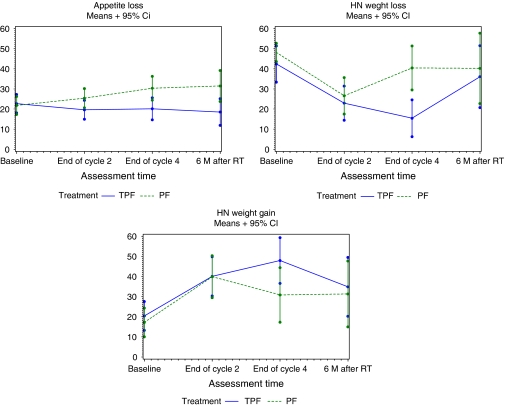
Evolution of mean scores in other non-selected scales over time.

An exploratory analysis of the non-preselected remaining HRQOL scales was performed. An increase in loss of appetite was noticed in the PF arm, whereas it was stable in the TPF arm (mean difference of 10.3; *P*=0.013 at the end of cycle 4); there was more weight loss (mean difference of 17.1; *P*=0.059) and less weight gain (mean difference of 25.1; *P*=0.0007) in the PF arm at the end of cycle 4 (Figure 3). Except for appetite loss, the measures were similar in the two treatment arms at 6 months after RT. For all other scales, there were no statistically significant treatment differences at any time point.

Analysis of the *ad hoc* pain thermometer data confirmed that there was no difference in pain intensity between the two treatment arms (data not shown). Evaluation of the clinician-assessed PSS-HN tool showed high compliance (75% at 6 months after RT), as these data were collected from case-report forms rather than HRQOL questionnaires. This tool provides the clinician's rating of performance status; an outcome related to, but not equivalent to QOL. Changes from baseline were analysed for the three items of this tool, that is, *Eating in public, Understandability of speech*, and *Normalcy of diet*, as an imbalance in baseline characteristics was noticed for two of the three scales.

**Figure 4:**
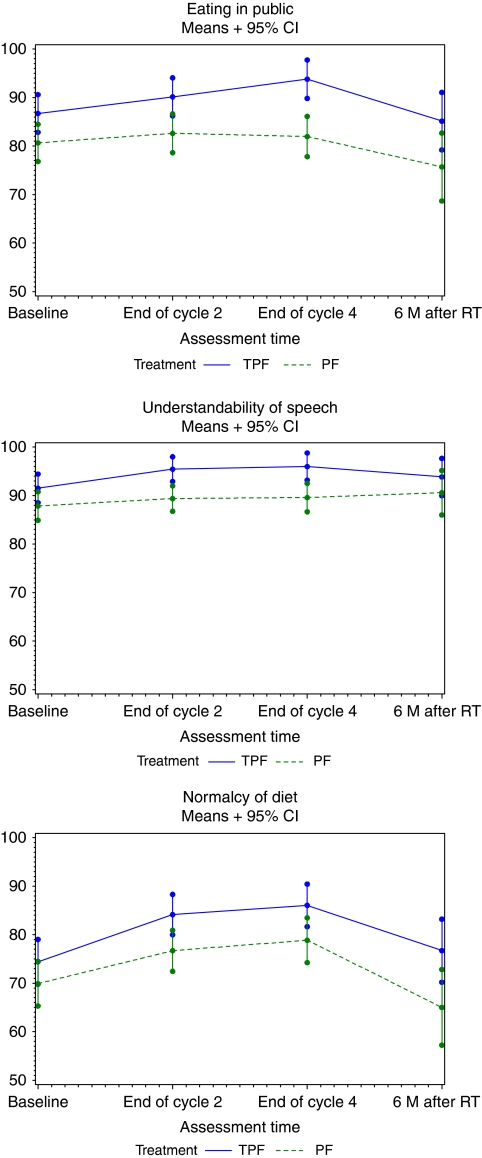
Evolution of mean scores from the Performance Status Scale for Head and Neck tool.

None of the comparison between arms for these scales reached the statistical significance. Compared with baseline, no major treatment differences were noted in these three scales (Figure 4).

## Discussion

In our randomised phase III study, HRQOL was assessed in patients with unresectable locoregionally advanced SCCHN after treatment with induction TPF or PF followed by RT.

The global HRQOL improved during induction CT in both treatment arms. As expected per protocol, no difference in global HRQOL was seen between arms during the treatment period. Interestingly, at 6 months after the end of the RT the global QoL remained higher than at baseline only in the TPF arm. In the PF arm, the global HRQOL returned to baseline scores, as usually seen in head and neck HRQOL studies (Abdel-Wahab *et al*, 2005; Curran *et al*, 2007). This resulted in a difference between the arms at 6 months after RT of 9.5 points, very close to a clinically meaningful improvement. Unfortunately, the compliance was too low to draw definite conclusions. In the pros, the compliance was similar in both arms. The imbalance between the number of patients still on study at this time point was mainly because of a higher rate of treatment discontinuation in the PF arm owing to toxicity and a higher rate of deaths owing to progressive disease, pointing towards a possible bias in disfavour of the experimental arm. In the cons, the analysis technique used relies on the assumption of data missing at random (MAR), which can always be criticised.

This trend towards an improvement in global HRQOL in the TPF arm occurred parallel to an increase in OS, higher response rate, and less severe toxicity, owing to a lower dose of cisplatin and 5-FU in the TPF arm than in the PF arm (Vermorken *et al*, 2007). Why HRQOL after 6 months was better in the TPF arm than in the PF arm is not completely clear, but can probably be explained, in part, by fewer recurrences of the tumour. Another explanation can be the lower dose of cisplatin and 5-FU used in TPF compared with PF. Long-term toxicity of cisplatin leads to polyneuropathy and ototoxicity, which can influence the global QoL. Patients’ overall HRQOL usually results from both treatment effects/side-effects and factors linked to the disease evolution, which are often indistinguishable.

A few investigators have assessed the longitudinal changes of HRQOL in patients with SCCHN during treatment. The general picture is a deterioration during the first 3 months after the start of treatment, followed by a slow recovery (de Graeff *et al*, 1999; Bairati *et al*, 2005; Fang *et al*, 2005). Locoregionally advanced disease patients included in the randomised trial of cetuximab with RT *vs* RT alone performed better in the combined arm (Bonner *et al*, 2006; Curran *et al*, 2007) and, although there was a gain in OS, no differences in HRQOL were observed. This study is the first reporting HRQOL during induction CT followed by RT, showing an improvement during the first weeks after start of neo-adjuvant CT. However, we did not measure the QoL during or in the last week of the RT. Thus, we can only speculate on the QoL during the RT in the TPF and PF arm. On the one hand, it could have been better in the TPF arm, because the trend in a better QoL, which was seen after the CT before the start of Rt, continued to improve, or on the other hand, it could have been worse in the TPF arm, because docetaxel can act as a radiosensitiser (Nabell and Spencer, 2003).

Swallowing dysfunction and aspiration are seen in a high proportion of patients with SCCHN after combined chemoradiation (Bentzen and Trotti, 2007). Therefore, swallowing and coughing, although not always related to aspiration, were selected as primary domains for this analysis. A trend to a higher reduction in swallowing and coughing problems was seen in the TPF arm compared with the PF arm, but the extent of the reduction was limited. In addition less loss of appetite was observed in the TPF arm, whereas less weight loss and more weight gain were observed in the TPF arm at the end of cycle 4. Eating problems may result from both the primary location of the head and neck cancer and treatment-induced adverse effects, such as pain in the mouth, problems with dentition, decreased saliva, and problems swallowing. Hence, weight loss is reported to affect 35–50% of patients with SCCHN, and is known to increase morbidity and mortality (van Bokhorst-de van der Schuer *et al*, 1999). Thus, the improvement of swallowing combined with less eating problems observed in the TPF arm is not only beneficial for HRQOL but probably causes less morbidity and mortality in the follow-up.

Our randomised controlled trial (RCT) had several limitations. Despite being a robust, well-designed, and monitored RCT, HRQOL compliance became very limited over time, making only analyses of short-term HRQOL data possible and not allowing to draw definite conclusions. However, this is not unexpected, as collecting data in head and neck trials can be difficult, and indeed, the lack of RCTs with HRQOL results in the literature may support this hypothesis. In addition, at the start of this study, not all translations of the EORTC Head and Neck module were available, hence reducing the amount of information available from the module. At last, even if, as per protocol, very precise timing for the HRQOL assessment was described, time windows need to be defined to perform the analysis and assign HRQOL data to the different time points and allow for some delays. A 3-week delay was allowed for the assessments ‘At the end of cycle 2’ and ‘At the end of cycle 4’, which may have caused a slight underestimation of the treatment effect.

Nevertheless, there are positive points. This was a RCT with a good sample size; a similar compliance in both arms; the use of a robust methodology under missing data of the MAR type; and no indication of a source of bias in the investigation of the missingness mechanism.

At this moment, the standard treatment of locally advanced SCCHN consists of concurrent chemoradiation. Concurrent chemoradiation was not incorporated as treatment in the EORTC 24971/TAX 323. However, at this moment, several studies are running with TPF as induction CT followed by concurrent chemoradiotherapy or RT combined with an inhibitor of the epidermal growth factor receptor (EGFR). Because these treatments cause more toxicity than RT without concurrent combination, an improvement of the QoL and swallowing after TPF would be of real value.

The field of treatment of the locally advanced SCCHN is moving quickly at this moment. The main goal of these developments is to administer a less toxic regimen to patients while keeping the same chance for cure. The use of intensity modulated RT (IMRT) and the use of targeted therapies, such as EGFR inhibitors, will lead to less toxicity and hopefully a better QoL for those patients (Feng *et al*, 2010). In addition, human papillomavirus-positive patients do have a better prognosis, both after CT and RT (Fakhry and Gillison, 2006). In future, these patients may be treated with a less-toxic regimen than the nowadays used concomitant chemoradiotherapy. The exact role in future for induction CT, that is, TPF, in this moving field is not yet clear. However, our observation of an improvement of global QoL during induction CT is important, and has to be investigated in future trials with induction CT followed by concurrent chemoradiation using IMRT, or followed by concurrent EGFR inhibition with RT.

In summary, in unresectable SCCHN patients, TPF compared with PF as induction CT before RT seemed to improve global HRQOL and swallowing in parallel with a significantly improved OS and less severe induced toxicity. These analogous improvements of a longer life with a better HRQOL in some areas can be seen as the ultimate goal of treatment of cancer patients and opens the door for further studies to determine the exact place of TPF as induction CT for the treatment of locally advanced SCCHN.

## Figures and Tables

**Table 1:** Compliance with QLQ-C30assessments

**Time points**		**Forms expected**	**Forms received**	**Percentage**	**Difference *P*-value**
Baseline		358	346	96.6	0.225
	TPF	177	169	95.5	
	PF	181	177	97.8	
End of cycle 2		328	282	86.0	0.340
	TPF	164	144	87.8	
	PF	164	138	84.2	
End of cycle 4		255	193	75.7	0.545
	TPF	136	105	77.2	
	PF	119	88	74.0	
At 6 months after RT^a^		198	108	54.5	0.794
	TPF	112	62	55.4	
	PF	86	46	53.5	
At 9 months after RT^a^		170	76	44.7	0.443
	TPF	95	40	42.1	
	PF	75	36	48.0	

Abbreviations: QLQ-C30=Quality of Life Questionnaire C30; TPF=docetaxel, cisplatin, and 5-fluorouracil regimen; PF=cisplatin and 5-fluorouracil regimen; RT=radiotherapy.

a 6 months and 9 months after the end of radiotherapy.

**Table 2:** Baseline QoL scores and reference data (Scott *et al*, 2008).

	**Reference data; head and neck cancer stage III–IV, mean (s.d.)**	**EORTC 24971 TPF arm (*N*=169), mean (s.d.)**	**EORTC 24971 PF arm (*N*=177), mean (s.d.)**	***P*-value for difference between arms**
*QLQ-C30*				
Global QoL	63.1 (22.4)	61.2 (23.3)	61.1 (20.3)	0.97
Physical functioning	81.2 (20.2)	85.7 (18.1)	88.8 (15.5)	0.09
Role functioning	78.8 (27.9)	79.2 (29.3)	84.1 (23.8)	0.09
Emotional functioning	71.2 (24.1)	70.2 (23.2)	75.0 (21.6)	0.05
Cognitive functioning	86.4 (19.1)	88.6 (17.7)	89.6 (15.8)	0.56
Social functioning	82.2 (24.7)	84.5 (23.0)	85.8 (21.8)	0.60
Fatigue	27.6 (25.0)	27.6 (25.7)	24.2 (22.7)	0.19
Nausea and vomiting	5.2 (13.3)	4.7 (12.6)	4.1 (12.7)	0.62
Pain	24.9 (26.3)	29.8 (26.4)	26.4 (23.0)	0.20
Dyspnoea	18.0 (26.6)	13.4 (22.5)	12.1 (21.5)	0.57
Insomnia	28.5 (32.4)	26.2 (29.4)	27.5 (31.0)	0.71
Appetite loss	19.4 (29.3)	22.9 (31.3)	21.6 (29.4)	0.69
Constipation	11.7 (23.2)	14.3 (24.4)	14.9 (26.5)	0.81
Diarrhoea	6.1 (16.7)	5.0 (15.8)	3.4 (10.7)	0.27
Financial difficulties	18.8 (30.2)	19.0 (29.0)	17.5 (27.6)	0.63
				
*QLQ-HN-35*				
Pain	29.9 (25.1)	29.6 (24.8)	27.4 (24.4)	0.51
Swallowing	27.5 (26.1)	26.1 (26.2)	28.3 (26.6)	0.51
Senses	20.0 (30.0)	9.8 (20.3)	12.3 (21.0)	0.34
Speech	27.1 (27.2)	20.6 (24.8)	23.3 (26.9)	0.43
Social eating	23.9 (26.7)	22.4 (26.1)	27.5 (30.7)	0.17
Social contact	13.2 (19.1)	10.3 (16.0)	12.0 (17.5)	0.44
Sexuality	32.3 (36.1)	32.4 (35.5)	24.8 (33.6)	0.11
Teeth	27.8 (35.0)	24.2 (34.6)	24.3 (36.5)	0.98
Opening mouth	22.4 (31.9)	28.5 (35.6)	22.6 (33.7)	0.19
Dry mouth	31.1 (34.2)	19.0 (25.9)	20.2 (25.1)	0.72
Sticky saliva	32.4 (35.4)	26.1 (34.4)	29.1 (31.3)	0.49
Coughed	34.9 (32.1)	30.8 (25.5)	30.5 (26.2)	0.92
Felt ill	21.7 (29.2)	17.1 (26.5)	19.1 (28.1)	0.58
Pain killers	52.8 (49.9)	58.1 (49.6)	59.0 (49.4)	0.90
Nutritional supplements	27.0 (44.4)	23.7 (42.7)	27.0 (44.6)	0.57
Feeding tube	18.3 (38.7)	14.4 (35.3)	13.8 (34.6)	0.89
Weight loss	41.3 (49.2)	42.7 (49.7)	52.2 (50.2)	0.15
Weight gain	25.9 (43.8)	20.9 (40.8)	17.2 (37.9)	0.48

Abbreviations: EORTC=European Organisation for Research and Treatment of Cancer; QOL=quality of life; QLQ-C30=Quality of Life Questionnaire C30; QLQ-HN-35=Head and Neck Cancer-Specific Module; TPF=docetaxel, cisplatin, and 5-fluorouracil regimen; PF=cisplatin and 5-fluorouracil regimen.

**Table 3:** QLQ-C30–global health status

**Assessment time**	**TPF**	**PF**	**Treatment difference *P*-value**
Baseline	61.5 (s.d.=1.69)	61.1 (s.d.=1.64)	0.8646
End of cycle 2	69.3 (s.d.=1.73)	66.8 (s.d.=1.75)	0.3290
End of cycle 4	70.7 (s.d.=1.91)	65.6 (s.d.=2.05)	0.0695
6 Months after RT	68.9 (s.d.=2.39)	59.4 (s.d.=2.73)	0.0092

Abbreviations: QLQ-C30=Quality of Life Questionnaire C30; TPF=docetaxel, cisplatin, and 5-fluorouracil regimen; PF=cisplatin and 5-fluorouracil regimen; RT=radiotherapy.

**Table 4:** Percentage of patients experiencing worsening/improvement from baseline during the follow-up period in selected scales

	**TPF (*N*=148)**	**PF (*N*=147)**	***P*-value for difference**
	***N* (%)**	***N* (%)**	
*Global quality of life* a			
⩾16.6 Points worsening	35 (23.6)	37 (25.2)	0.79
⩾25 Points worsening	21 (14.2)	21 (14.3)	1.00
⩾16.6 Points improvement	80 (54.1)	69 (46.9)	0.25
⩾25 Points improvement	52 (35.1)	40 (27.2)	0.17
			
*Coughing* b			
⩾33.3 Points worsening	19 (12.8)	24 (16.3)	0.41
⩾33.3 Points improvement	49 (33.1)	35 (23.8)	0.09
			
*Pain* c			
⩾11.1 Points worsening	29 (19.6)	21 (14.3)	0.28
⩾22.2 Points worsening	19 (12.8)	13 (8.8)	0.35
⩾11.1 Points improvement	50 (33.8)	42 (28.6)	0.38
⩾22.2 Points improvement	40 (27.0)	24 (16.3)	0.03
			
*Speech problems* d			
⩾11.1 Points worsening	34 (23.0)	24 (16.3)	0.19
⩾22.2 Points worsening	17 (11.5)	15 (10.2)	0.85
⩾11.1 Points improvement	51 (34.5)	43 (29.3)	0.38
⩾22.2 Points improvement	35 (23.6)	27 (18.4)	0.32
			
*Swallowing* e			
⩾11.1 Points worsening	18 (12.2)	27 (18.4)	0.15
⩾22.2 Points worsening	12 (8.1)	16 (10.9)	0.43
⩾11.1 Points improvement	47 (31.8)	39 (26.5)	0.37
⩾22.2 Points improvement	31 (20.9)	32 (21.8)	0.89

Abbreviations: TPF=docetaxel, cisplatin, and 5-fluorouracil regimen; PF=cisplatin and 5-fluorouracil regimen.

a Global QoL score may take all values from 0 to 100 distant by 8.3 points (0, 8.3, 16.6, and so on). A shift of more than 10 points means a shift of 16.6 points or more. A shift of more than 20 points means a shift of 25 points or more.

b Coughing score may take all values from 0 to 100 distant by 33.3 points (0, 33.3, 66.6, and so on).

c Pain score may take all values from 0 to 100 distant by 2.8 points.

d Speech score may take all values from 0 to 100 distant by 5.5 points.

e Swallowing score may take all values from 0 to 100 distant by 2.8 points.
